# Fluid cognitive ability is a resource for successful emotion regulation in older and younger adults

**DOI:** 10.3389/fpsyg.2014.00609

**Published:** 2014-06-17

**Authors:** Philipp C. Opitz, Ihno A. Lee, James J. Gross, Heather L. Urry

**Affiliations:** ^1^Department of Psychology, Tufts UniversityMedford, MA, USA; ^2^Department of Psychology, Stanford UniversityStanford, CA, USA

**Keywords:** emotion regulation, cognitive reappraisal, working memory, cognitive ability, SOC-ER, older adults

## Abstract

The Selection, Optimization, and Compensation with Emotion Regulation (SOC-ER) framework suggests that (1) emotion regulation (ER) strategies require resources and that (2) higher levels of relevant resources may increase ER success. In the current experiment, we tested the specific hypothesis that individual differences in one internal class of resources, namely cognitive ability, would contribute to greater success using cognitive reappraisal (CR), a form of ER in which one reinterprets the meaning of emotion-eliciting situations. To test this hypothesis, 60 participants (30 younger and 30 older adults) completed standardized neuropsychological tests that assess fluid and crystallized cognitive ability, as well as a CR task in which participants reinterpreted the meaning of sad pictures in order to alter (increase or decrease) their emotions. In a control condition, they viewed the pictures without trying to change how they felt. Throughout the task, we indexed subjective emotional experience (self-reported ratings of emotional intensity), expressive behavior (corrugator muscle activity), and autonomic physiology (heart rate and electrodermal activity) as measures of emotional responding. Multilevel models were constructed to explain within-subjects variation in emotional responding as a function of ER contrasts comparing increase or decrease conditions with the view control condition and between-subjects variation as a function of cognitive ability and/or age group (older, younger). As predicted, higher fluid cognitive ability—indexed by perceptual reasoning, processing speed, and working memory—was associated with greater success using reappraisal to alter emotional responding. Reappraisal success did not vary as a function of crystallized cognitive ability or age group. Collectively, our results provide support for a key tenet of the SOC-ER framework that higher levels of relevant resources may confer greater success at emotion regulation.

## Introduction

Emotions are frequently helpful in achieving adaptive goals. However, our emotions can at times be either the wrong intensity or duration, thereby impeding rather than facilitating goal achievement. When this happens, it is useful to regulate our emotions. Gross' (1998) process model of emotion regulation (ER) describes five families of ER strategies, namely, situation selection, situation modification, attentional deployment, cognitive change, and response modulation. Research has shown that people differ with regard to the strategies they habitually choose to regulate their emotions (Gross and John, 2003) and there are individual differences in success at implementing specific ER strategies as well (for example, as a function of older vs. younger age; Shiota and Levenson, 2009; Opitz et al., 2012a).

Although ER has been linked to important adaptive outcomes (Appleton et al., 2013; Gross, 2014), we know very little about the factors that explain these individual differences. The Selection, Optimization, and Compensation with Emotion Regulation (SOC-ER) framework (Urry and Gross, 2010; Opitz et al., 2012b) offers a set of factors that may help explain individual differences in ER.

According to the SOC-ER framework, successful ER depends on three factors: (1) choosing ER strategies for which resources are available at sufficient levels (Selection), (2) devoting time, practice, and effort to using these strategies successfully (Optimization), and (3) choosing alternate ER strategies when the ER strategy one chose initially is unsuccessful, perhaps as a result of insufficient resources (Compensation) (Urry and Gross, 2010; Opitz et al., 2012b). The cornerstone of this framework is the notion that successful ER requires resources—internal abilities and/or environmental affordances that promote the use of a given ER strategy. From this perspective, as resource levels vary within individuals (i.e., from one situation to the next, or from one phase of the life span to the next) or across individuals (i.e., from one person to another), ER success should vary accordingly. In the present study, we test this cornerstone SOC-ER idea with respect to individual differences in one class of resources—cognitive ability—and one ER strategy, cognitive reappraisal (CR).

CR refers to altering one's emotional response by reinterpreting the emotion-eliciting situation or one's response to it. Neuroimaging studies have shown that successful CR recruits brain areas involved in working memory, suggesting that successful CR may depend on the ability to hold information in mind (Ochsner et al., 2002, 2004; McRae et al., 2010). Additionally, CR has been shown to be most effective when initiated early in the emotion-generative cycle (Sheppes and Meiran, 2007), signifying that the ability to process information quickly may be beneficial, too. These observations hint at two cognitive abilities—working memory capacity (WMC) and processing speed (PS)—that may be resources for CR and thus contribute to CR success. Providing partial support for this idea, cross-sectional research suggests that older adults (OA), who exhibit deficits in WMC and PS (e.g., Salthouse et al., 2003; Hedden and Gabrieli, 2004), are less successful using some forms of CR compared to younger adults (YA), like detached reappraisals aimed at decreasing negative emotions (Shiota and Levenson, 2009). Of course, the observation of an age difference provides only indirect support for the role of cognitive ability as a resource for CR. To clarify our understanding of the relationship between these putative resources and CR, research directly assessing relations between cognitive abilities and CR success is needed.

Schmeichel et al. (2008) presented the first direct assessment of the role of cognitive ability as a resource for successful CR in a between-subjects design. These authors demonstrated that higher levels of WMC were correlated with reduced experience of unpleasant emotion in a group of participants who were assigned to adopt neutral, nonemotional appraisals of disgusting film clips. In another between-subjects design, Malooly et al. (2013) assessed relations between affective and nonaffective cognitive flexibility and successful CR. In both cases, cognitive flexibility was operationalized as reaction time costs of switching between rules regarding how to process different features of target stimuli. In the nonaffective flexibility task, neither the rules nor the target stimuli were emotional; in the affective flexibility task, both were emotional. Results of this study suggested that higher levels of affective cognitive flexibility were associated with reduced experience of sad emotion in a group of participants who were assigned to reappraise sad film clips by adopting a neutral, analytic, objective mindset. Finally, McRae et al. (2012) examined the relationship between CR and WMC, as well as other components of cognitive ability, in a within-subjects context. Results indicated moderate positive correlations between the ability to use CR to decrease unpleasant emotions and WMC and set-shifting costs; a similar trend was observed for perceptual reasoning. By contrast, verbal fluency and response inhibition were uncorrelated with reappraisal ability.

Together, these findings from Schmeichel et al. (2008); McRae et al. (2012); Malooly et al. (2013) and provide convergent, direct support for the role of cognitive ability—particularly, WMC and cognitive flexibility—as a resource for successful CR. These studies further suggest that not all cognitive abilities are resources for CR, pointing to a certain degree of specificity regarding the role of cognitive resources in CR. Still, with only these three studies in hand, important questions remain.

In the present paper, our primary goal is to address the following questions: First, are as-of-yet unexamined cognitive abilities resources for successful CR? Here, we examine PS, an index of cognitive ability that also has promise as a resource for CR. We also examine working memory, verbal ability, and perceptual reasoning to conceptually replicate previous findings. Second, are associations between cognitive ability and CR success dependent on whether one's CR goal is to decrease or increase one's emotional response? The two previous studies found that some aspects of cognitive ability were associated with the ability to use CR to *decrease* unpleasant emotional experience. Here we examine the ability to *increase* unpleasant emotion too. This is important methodologically because it provides an active, effortful comparison. In addition, this is important conceptually since ER efforts are sometimes directed in pursuit of contrahedonic feeling states (Ford and Tamir, 2012; Tamir and Ford, 2012; Tamir et al., 2013).

To achieve our primary goal, we recruited both younger and OA. In light of aforementioned age differences in cognitive abilities of interest, recruiting younger and OA allowed us to maximize meaningful variation in cognitive abilities in our sample. This also allowed us to address a secondary goal, which was to assess whether older age—normatively associated with decrements in cognitive resources including WM and PS—impacts CR success. Previous research has demonstrated mixed results, with some indicating age-related decrements for reappraisals aimed at decreasing unpleasant emotions (Opitz et al., 2012a) and detached reappraisals (Shiota and Levenson, 2009), and others indicating age-related sparing for reappraisals aimed at increasing unpleasant emotions (Opitz et al., 2012a) and positive reappraisals (Shiota and Levenson, 2009; Lohani and Isaacowitz, 2013). However, these mixed results may reflect variation across studies in emotional load and task demands. OA may perform less well only when CR tasks include highly potent negative images and require excessive shifting from one condition to the next. Here we examine CR success in a task that keeps emotional load and task demands to a minimum.

In pursuit of our primary and secondary goals, our younger and older participants completed well-validated, standardized neuropsychological measures of fluid and crystallized cognitive ability and a CR task. In the CR task, participants used CR to increase or decrease their emotional response to sad pictures. We chose sad stimuli specifically as previous research suggests sadness to be among the most frequently regulated emotions (Gross et al., 2006), thus maximizing the likelihood that participants would want to regulate emotions elicited by the pictures and have some experience doing so. These two conditions were compared to a view control condition in which participants responded naturally to the pictures without trying to change how they felt. We used a gaze-directed variant of the CR task validated in previous studies (Urry, 2010; Opitz et al., 2012a). The gaze direction manipulation enabled us to maximize the extent to which deployment of attention to arousing and non-arousing information was equivalent across the increase, view, and decrease CR instructions. This may be particularly important when studying OA because OA are more apt to deploy greater attention to positive than negative emotional information in some contexts (Isaacowitz et al., 2008, 2009).

During the CR task, we recorded subjective emotional experience (self-reported ratings of intensity), expressive behavior (corrugator muscle activity), and autonomic physiology [heart rate (HR) and electrodermal activity (EDA)] as measures of emotional responding. Multilevel models were constructed to explain within-subjects variation in emotional responding as a function of the CR manipulations and between-subjects variation as a function of cognitive ability or age. Our comprehensive approach offers several benefits. First, we are capturing trial-by-trial variation in emotional responding in three components of emotion. Past studies suggest that components of emotional responding cohere to some degree (Mauss et al., 2005), but this coherence is imperfect. This means one cannot measure just one component and make strong inferences about other, unmeasured components. This also means some components of emotional responding may show predicted effects while others may not. Second, our measures of expressive behavior and autonomic physiology are less subject to demand characteristics than self-report evaluations of emotional experience. Thus, were we to find predicted effects for expressive behavior and/or autonomic physiology, this would bolster the interpretation that differences between the CR conditions of interest reflect emotion regulatory success and not just demand characteristics of the experiment. Third, the continuous nature of our peripheral physiological measures provided a moment-to-moment index of responses during the task, allowing us to separately model pre-instruction and post-instruction activity. This allowed us to account for initial emotion reactivity (for which there often are age differences).

With these design features in place, we tested the hypothesis that higher levels of fluid (perceptual reasoning, PS, and working memory) and perhaps crystallized (verbal ability) cognitive abilities would predict greater CR success.

## Methods

### Participants

Thirty younger (20 female, 18–22 years, *M* = 19.5, *SD* = 1.18) and 30 older (17 female, 55–71 years, *M* = 61.9, *SD* = 5.14) adults were recruited via internet (e.g., http://www.craigslist.com), local newspaper, and community e-mailing list advertisements. Participants endorsed the following non-exclusive racial categories: Asian: 11 (18.3%); White: 46 (76.7%); Declined to respond: 3 (5%). Two participants (3.4%) endorsed being of Hispanic origin, and two participants declined to report ethnicity. See Table 1 for additional participant characteristics.

**Table 1:** **Characteristics of the sample**.

**Measure**	**Younger**	**Older**	**Significant age difference?**
Mean (*SD*) age in years	19.45 (1.18)	61.90 (5.14)	*t*_(30.96)_ = −42.44, *p* < 0.001
Total *N*	30	30	
Men	10 (33%)	13 (43%)	χ^2^(1) = 0.648, *p* = 0.421
Women	19 (67%)	16 (57%)	
**HIGHEST LEVEL OF EDUCATION**			
High school diploma	11 (36%)	1 (3%)	χ^2^(3) = 30.21, *p* < 0.001
Some college	16 (53%)	6 (20%)	
College diploma	2 (6%)	10 (33%)	
Graduate degree	0	12 (40%)	
**MARITAL STATUS**			
Never married	29 (97%)	3 (10%)	χ^2^(4) = 47.13, *p* < 0.001
Married	0	12 (40%)	
Separated	0	1 (3%)	
Divorced	0	11 (36%)	
Widowed	0	2 (6%)	

Demographic data for two participants (one younger) were not collected, and are therefore not included (except in total N).

No participants reported any history of psychiatric or neurological disorders, or current use of any psychoactive medications. Before completing the experiment, participants completed a hearing test (WWW Hearing Test, Digital Recordings, Halifax, NS, Canada) and two vision tests (Arditi, 2005; Dougherty et al., 2005) to ensure they could hear and see the stimuli. All procedures were approved by the Social, Behavioral, and Educational Research Institutional Review Board at Tufts University. Participants provided written informed consent prior to participating.

### Materials

#### Cognitive ability

To assess cognitive ability, participants completed four subtests of the Wechsler Adult Intelligence Scale IV (WAIS IV, Pearson, San Antonio, TX).

We assessed working memory using the Digit Span (DS) task, in which participants hear and verbally reproduce an increasing number of digit strings (two trials per digit string length) in forward order (DS Forward), backward order (DS Backward), or in ascending order (Sequencing). The task continues until both trials of a given digit string length are incorrect or until the last item of the task is reached.

We assessed verbal ability using the Vocabulary (VC) task, in which participants provide definitions for increasingly difficult words presented orally. For instance, on an early (easy) trial, participants defined “APPLE,” whereas on a later (hard) trial, participants defined “PALLIATE.” For the purposes of this paper, we report scores based on one half of the VC task. The other difficulty-matched half was subject to a manipulation of no relevance to this paper.

We assessed perceptual reasoning using the Block Design (BD) task, in which participants are given a set of nine red-and/or-white cubes, which they have to assemble to reproduce two-dimensional patterns of increasing difficulty. The task continues until two consecutive trials are incorrect or until the last item of the task is reached.

Lastly, we assessed PS using the Coding (CD) task, in which participants are given a sheet of paper containing a “key” that links numbers one through nine with simple non-alphanumeric symbols. Participants are asked to draw the correct symbol (according to the key) below each of multiple rows of numbers on the sheet below until 2 min has elapsed or until the last item of the task is reached. Data for one older participant were not available for CD due to participant misunderstanding of instructions.

OA tend to perform less well than YA on the DS, BD, and CD subtests, which are indicators of fluid cognitive ability; OA perform similarly or better than YA on the VC subtest, an indicator of crystallized cognitive ability (Salthouse et al., 2003; Hedden and Gabrieli, 2004).

#### Picture stimuli

In the CR task, which is described below, participants viewed a set of 120 digital color pictures (800 × 600 pixels). Most were selected from the International Affective Picture System (IAPS; Lang et al., 2008) using normative valence ratings provided by Mikels et al. (2005). Specifically, we used those images that Mikels et al. (2005) categorized as sad on a scale from 1 to 7, where 1 corresponded to *not at all* sad, and 7 corresponded to *very sad*; our set was moderately sad on average (*M* = 4.09, *SD* = 0.85). To have enough sad pictures to avoid repetition, we supplemented the IAPS pictures with pictures selected from stock photo libraries (Shutterstock and the Wellcome Image Collection). These pictures, for which no normative ratings exist, were selected for content similar to those selected from the IAPS. The final set of 120 sad pictures mostly depicted withdrawn, lonely, or sad older and YA and/or injured, solitary, or apparently sad animals. We purposefully sought content that is relevant to participants in both age groups.

#### Cognitive reappraisal task

The pictures were presented in the context of a gaze-directed CR task, a variant of the task validated by Urry (2010) and also used with both younger and OA by Lang et al. (2008); Opitz et al. (2012b). This task comprised 120 trials described by crossing two within-subjects factors, CR condition (increase, view, decrease, no CR) and gaze direction condition (arousing, free/no gaze direction, non-arousing). Prior to completing the CR task, participants received in-depth training to ensure that they understood the CR and gaze direction manipulations (described below) as well as how to rate the intensity of their emotional response on each trial.

For the CR manipulation, participants were trained to increase or decrease their emotional response to the pictures by considering the personal relevance of the depicted situation (self-focused reappraisal) or imagining alternative outcomes (situation-focused reappraisal). Cues to begin using CR were presented via single-word audio recording, “increase” or “decrease” 3 s after picture onset. As part of their training, participants saw a picture of a man lying in a hospital bed, seemingly in pain. For the “increase” instruction, participants were told they could imagine that “you yourself or a loved one are the person in this picture, or that you are present and are witnessing the man's pain and struggle.” Conversely, for the “decrease” instruction, participants were told that they could imagine that “you are simply observing the man objectively, or that he will get better soon.” A third audio recording, “view,” served as a cue to respond naturally without trying to change how they felt. A fourth CR condition, “no CR,” entailed viewing the picture with no instruction, which enabled us to determine whether the simple instruction to “view” would alter emotional responding. For all four conditions, participants were instructed not to think of the pictures as fake or unreal.

For the gaze direction manipulation, participant gaze was directed to an emotionally arousing or non-arousing (neutral) area of the picture beginning 4 s after picture onset, or participant gaze was not directed, i.e., allowed to vary freely. This gaze direction manipulation enabled us to control the ways in which participants deployed their visual attention to the emotional information in the pictures during the 8-s regulation period of interest (c.f., Isaacowitz et al., 2008, 2009). As validated in prior work (Urry, 2010), eye tracking data (not shown) confirmed that the present sample of participants followed the gaze direction instruction equally across the CR conditions, increasing looking time in the designated areas of interest following the manipulation onset. These data also suggested that older and YA did not differ in their gaze behavior.

**Figure 1:**
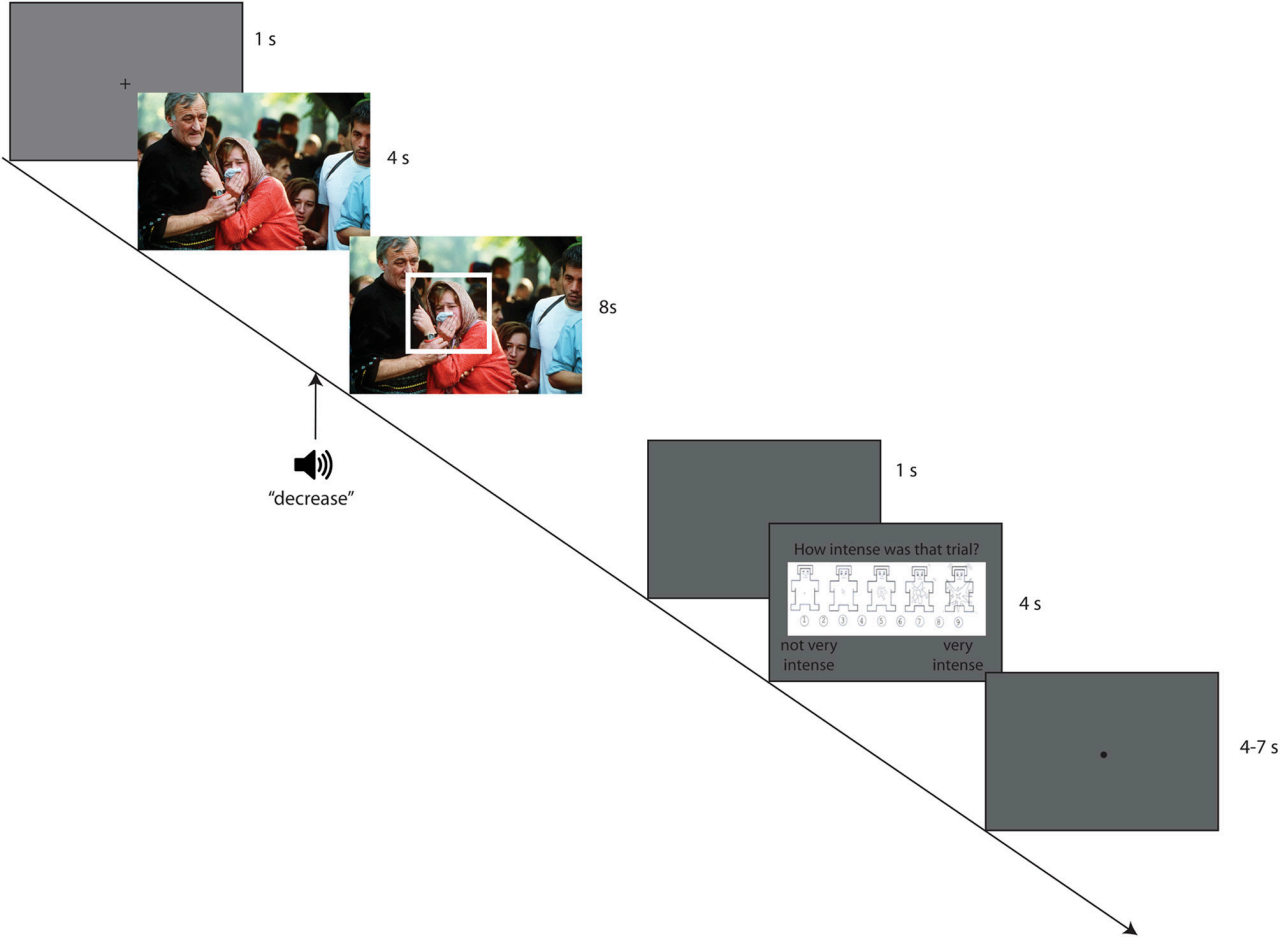
**Trial structure**. Participants first saw a fixation cross for 1 s, followed by a picture for a total of 12 s. Three seconds after picture onset, participants heard the cognitive reappraisal (CR) instruction for that trial, if applicable (in this case, “decrease”). One second thereafter, a box directed them to look in an arousing or nonarousing area of the picture, if applicable (in this case, the box is directing gaze to an arousing area). After the 8-s regulatory period during which they followed the CR and gaze direction instructions, participants briefly saw a gray screen (1 s). This was followed by a 9-point intensity rating screen which was available for up to 4 s. After the rating screen, a gray screen with a black dot signaled a break between trials, which varied from 4 to 7 s. Photo of mourning family from Evstafiev (1992). Used with permission of the photographer.

The CR task was programmed using E-Prime version 1.2.1.8 (Psychology Software Tools, Inc., Sharpsburg, PA) in 10 blocks of 12 trials each. Pictures were presented on a TFT display (1280 × 1204 maximum resolution) while auditory instructions were delivered via speakers in a sound- and RF-shielded experiment booth. Half of the blocks included the “increase,” “view,” and “no CR” conditions, while the other half included the “decrease,” “view,” and “no CR” conditions. We blocked increase and decrease CR trials in this manner to reduce the mental set shifting demands of the experiment. CR conditions, gaze directions, and pictures were otherwise randomly assigned to each trial. The gaze-directed CR task trial structure is depicted in Figure 1.

### Dependent variables

Three measures of emotional responding were recorded during the CR task. Self-report ratings of emotional intensity were collected at the end of each trial. Expressive behavior (corrugator muscle activity via facial electromyography) and autonomic physiology (HR and EDA) were collected continuously using an MP150 system (Biopac, Goleta, CA) and processed offline using ANSLAB (Wilhelm and Peyk, 2005).

#### Self-report ratings of intensity

Self-report ratings of emotional intensity ranging from 1 (not very intense) to 9 (very intense) were collected at the end of each trial. This 9-point numeric scale was illustrated with five figures based on the arousal scale of the Self-Assessment Manikin (Lang et al., 2008). Figures ranged from a relaxed, sleepy figure at the left (not at all intense) to an excited, wide-eyed figure at the right (very intense; see Figure 1).

#### Corrugator electromyography (EMG)

Corrugator activity has been shown to be sensitive to stimulus valence, with greater activity being associated with higher levels of stimulus unpleasantness (Bradley and Lang, 2007). Two 4-mm Ag/AgCl electrodes were placed in bipolar configuration over the right eye per Fridlund and Cacioppo (1986). One ground was placed on the forehead. The data were sampled at 1000 Hz and bandpass filtered online (5 Hz to 3 kHz; 60-Hz notch filter on). Offline, data were resampled to 400 Hz, rectified, filtered (16 Hz low-pass), decimated to 4 Hz, and smoothed (1-s prior moving average).

#### Electrocardiography

Electrocardiography was used to measure HR, which is dually innervated by the sympathetic and parasympathetic branches of the autonomic nervous system. In event-related paradigms involving passive viewing of negative pictures, HR is sensitive to stimulus valence and often exhibits an initial, parasympathetically mediated deceleration (Bradley and Lang, 2007). Two disposable Ag/AgCl electrodes pregelled with 7% chloride gel (1 cm circular contact area) were placed under the left and right collarbones on the chest after swabbing with an alcohol or an electrode prep pad. ECG was acquired continuously at 1000 Hz. Offline, the ECG signal was downsampled to 400 Hz and bandpass-filtered from 0.5 to 40 Hz.

Interbeat interval series were created by identifying R-spikes using automated ANSLAB algorithms. R-spikes that were not detected automatically, thus leading to an erroneously long period between successive R-spikes, were marked for inclusion by hand. Similarly, R-spikes that were identified incorrectly, thus leading to an erroneously short period between successive R-spikes, were removed by hand. Following such artifact correction, the interbeat interval series was converted to HR in beats per minute. HR data were decimated to 10 Hz and then smoothed with a 1-s prior moving average filter.

#### Electrodermal activity

EDA was selected as a pure measure of sympathetic activation of the autonomic nervous system. Two disposable Ag/AgCl electrodes pregelled with 0.5% chloride isotonic gel (1 cm circular contact area) were attached to the distal phalanges of the index and middle fingers on the left hand. EDA level was recorded with DC coupling and constant voltage electrode excitation at 31.25 Hz (sensitivity = 0.7 nS). Offline, EDA was smoothed with a 1-Hz, low-pass filter, decimated to 10 Hz, and linearly detrended on a trial-by-trial basis. One ground electrode for all physiological channels was placed on the forehead.

### Procedures

After providing informed consent, participants completed the BD, CD, DS, and VC subtests of the WAIS-IV in the order described above. Participants were then prepared for physiological recording and completed the training procedure for the CR task. Immediately following this training, participants completed the CR task. At various points in the session, participants completed additional questionnaires and a second set of WAIS-IV subtests. These additional procedures were not the focus of this report and some were subject to an unrelated manipulation thus the data are not reported here^1^.

### Data reduction, retention, and analysis

For the continuous peripheral physiological measures, we summarized raw activity for two periods of interest in each trial, pre-instruction activity (reactivity period; mean of activity occurring 4 s after picture onset) and post-instruction activity (regulation period; mean of activity occurring 8 s after both ER manipulations). To prevent leveraging of condition estimates by outlying values in the dependent variables, trials for which the estimate of Mahalanobis Distance within each measure was too large (*p* < 0.001) were set to missing (see Fidell and Tabachnick, 2003). An average of 95.4% (*SD* = 0.789; minimum = 94.2%, maximum = 96.0%) of all observations were available for analysis.

Multilevel analyses with two levels, some incorporating latent variables (see Hox, 2002; Kline, 2010), were conducted using Mplus v. 7.11 (Muthén and Muthén, 1998–2013). Continuous predictors were grand-mean centered. Estimates were generated (and missing values handled) using full information maximum likelihood estimation with robust standard errors. Although multilevel analyses are reported less often than traditional repeated measures analyses, they have three key advantages. First, this approach allows us to model emotional responding as a latent construct with experiential (self-reported intensity), expressive (corrugator activity), and autonomic physiological (HR, EDA) components. Traditional analyses compel one to analyze each measure as if they were independent. Second, this approach allows us to model trial-related variation in the dependent variable within each condition. Traditional analyses compel one to average across trials within each condition with the perhaps unfounded assumption that there is no trial-related variation. Third, this approach provides estimates using all available data. Traditional analyses compel listwise deletion of cases with missing observations.

## Results

Results are presented below in four sections. We first present preliminary analyses undertaken to determine (1) whether there are individual differences in pre-instruction emotion reactivity, and (2) whether there are main and/or interactive effects of our CR and gaze direction manipulations. Then, in pursuit of our primary goal, we present analyses germane to determining whether cognitive ability predicts successful CR. Next, in pursuit of our secondary goal, we present analyses germane to determining whether there are age differences in CR success. Finally, we report supplemental analyses that provide estimates of emotional responding as a function of age group, CR, and gaze direction during the regulation period on a measure-by-measure basis.

### Preliminary analyses

#### Individual differences in pre-instruction emotion reactivity

Using a two-level structural equation model, we examined whether there were individual differences in pre-instruction reactivity to the stimuli as a function of between-subjects variables of primary and secondary interest in this research (fluid cognitive ability, crystallized cognitive ability, age group). We also examined education level and marital status, both of which varied as a function of age group and thus represent control variables of interest.

On the first (within-subjects) level, activity during the reactivity period was regressed on baseline activity (mean of activity occurring 1 s prior to picture onset) for each of the three physiological measures.

On the second (between-subjects) level, activity during the reactivity period for each measure (self-reported intensity corrugator activity, HR, and EDA) was regressed on five between-subjects predictors as follows: fluid cognitive ability, crystallized cognitive ability, age group (older = 1, younger = 0), ever married (1 = yes, 0 = no), and education level. Fluid cognitive ability was modeled as a latent predictor variable with three manifest indicators, BD, CD, DS; variance was constrained at 1. Crystallized cognitive ability was modeled as the stand-alone manifest indicator, VC.

Participants exhibited significant, nonzero values for all four criterion variables (self-reported intensity *B* = 4.26, *SE* = 0.353; corrugator activity *B* = 7.15, *SE* = 0.459; HR *B* = 72.1 *SE* = 0.876; and EDA *B* = 11.57, *SE* = 0.014, all *p*s < 0.001). Factor loadings for the fluid cognitive ability latent predictor variable were positive and significant (*p* = 0.001 for BD and CD; *p* = 0.002 for DS). Finally, there were some significant individual differences in the criterion variables as follows: Higher fluid cognitive ability and lower crystallized cognitive ability were associated with lower EDA (*B* = −0.02, *SE* = 0.009, *p* = 0.031 and *B* = 0.002, *SE* = 0.001, *p* = 0.031, respectively) but not self-reported intensity, corrugator activity, or HR (all *p*s > 0.20). Older age was associated with lower corrugator activity (*B* = −1.63, *SE* = 0.782, *p* = 0.037) but not self-reported intensity, HR, or EDA (all *p*s > 0.70). Ever having been married was associated with higher corrugator activity (*B* = 1.37, *SE* = 0.451, *p* = 0.002), lower HR (*B* = −3.28, *SE* = 1.557, *p* = 0.035), and lower EDA (*B* = −0.06, *SE* = 0.027, *p* = 0.026) but not self-reported intensity (*p* = 0.763). Education level was not associated with any of the criterion variables (all *p*s > 0.17). We do not consider these isolated and non-predicted findings further.

#### Main and interactive effects of CR and gaze direction

In a second two-level structural equation model, we tested the main and interactive effects of the CR and gaze direction manipulations on within-subjects (trial-by-trial) variation in emotional responding. The first column of Table 2 summarizes the criterion and predictor variables in this preliminary analysis.

**Table 2:** **Parameter estimates from preliminary analysis examining cognitive reappraisal and gaze direction effects on emotional responding**.

**Emotion response latent criterion variable**	***B* (*SE*)**
**Indicators**	**Factor loadings**
Self-reported intensity	0.961 (0.279)^*^
Corrugator activity	0.508 (0.085)^**^
Heart rate	0.077 (0.1)
Skin conductance	0.003 (0.001)^+^
**Within-subjects predictors**	***B* (*SE*)**
**CR CONTRASTS**	
Decrease—view^a^	−0.637 (0.171)^**^
Increase—view^a^	0.997 (0.237)^**^
No CR—view	−0.176 (0.063)^*^
**GAZE DIRECTION CONTRASTS**	
Arousing—not directed^a^	0.07 (0.04)^+^
Non-arousing—not directed^a^	−0.166 (0.069)^*^
**CR × GAZE DIRECTION INTERACTION CONTRASTS**	
(Decrease—view) × (Arousing—not directed)	−0.112 (0.07)
(Increase—view) × (Arousing—not directed)	0.051 (0.066)
(No CR—view) × (Arousing—not directed)	−0.021 (0.062)
(Decrease—view) × (Non-arousing—not directed)	0.197 (0.079)^*^
(Increase—view) × (Non-arousing—not directed)	−0.045 (0.061)
(No CR—view) × (Non-arousing—not directed)	−0.037 (0.06)

CR, cognitive reappraisal. Pre-instruction activity was a positive, significant predictor of post-instruction activity for all three physiological measures; these estimates are not reported for the sake of brevity.

a Slope was estimated as a random effect.

** *p < 0.001*,

* *p < 0.05*,

+ *p < 0.10*.

On the first level, activity during the regulation period was regressed on activity during the reactivity period for each physiological measure in order to isolate CR- and gaze direction-related variation in emotional responding. Emotional responding was then modeled as one latent dependent variable with variance constrained at 1. Emotional responding had four manifest indicators, namely self-reported emotional intensity and corrugator activity, HR, and EDA during the regulation period.

The emotional responding latent dependent variable was regressed on three CR condition contrasts [increase (1) vs. view (−1), decrease (1) vs. view (−1), and no CR (1) vs. view (−1)] to estimate the effects of CR, and two gaze direction condition contrasts [arousing (1) vs. not directed (−1) and non-arousing (1) vs. not directed (−1)] to estimate the effects of gaze direction. The latent variable was also regressed on six contrasts reflecting the products of each of the CR condition contrasts with each of the gaze direction condition contrasts to estimate the interaction between CR and gaze direction.

On the second level, random slopes were estimated for the decrease and increase CR predictors and the arousing and non-arousing gaze direction predictors from the first level. Random slopes were not estimated for the no CR predictor, which was a covariate of no interest, or for the six interaction contrasts by convention (Hoffman and Rovine, 2007).

As shown in the second column of Table 2, this analysis revealed that two of four manifest indicators, self-reported intensity and corrugator activity, loaded positively and significantly on the latent emotional response dependent variable on the first level (both *p* = 0.001); a third, skin conductance, exhibited a trend in the same direction (*p* = 0.083). In addition, the CR manipulation impacted emotional responding as expected. Participants responded with greater emotion in the increase condition vs. the view condition (*B* = 1.00, *p* < 0.001), lower emotion in the decrease condition vs. the view condition (*B* = −0.64, *p* < 0.001), and lower emotion in the no CR condition vs. the view condition (*B* = −0.18, *p* = 0.006).

The gaze direction manipulation also impacted emotional responding. Participants responded with greater emotion when gaze was directed to an arousing area vs. when gaze was not directed (*B* = 0.07, *p* = 0.086, a nonsignificant trend), and lower emotion when gaze was directed to a non-arousing area vs. when gaze was not directed (*B* = −0.17, *p* = 0.016). Importantly, gaze direction moderated the decrease CR effect, significantly for the non-arousing gaze direction contrast (*B* = 0.20, *p* = 0.013) but not significantly for the arousing gaze direction contrast (*B* = −0.11, *p* = 0.108). Neither gaze direction contrast moderated the increase CR effect (both *p* > 0.40).

In light of the significant interaction above, we conducted a set of follow-up analyses in which we estimated the three CR effects separately for each of the three gaze direction conditions. In these analyses, a random slope was estimated at the between-subjects level for the decrease CR contrast. When gaze was not directed, participants responded with lower emotion in the decrease condition vs. the view condition (*B* = −0.42, *p* = 0.009). When gaze was directed to an arousing area, this decrease CR effect was even stronger (*B* = −0.98, *p* < 0.001). However, when gaze was directed to a non-arousing area, the decrease CR effect narrowly reached significance in the opposite direction (*B* = 0.35, *p* = 0.048).

These results suggest that CR generally impacts emotional responding in expected ways. However, using CR to decrease emotional responding was effective only when participants were directing attention to emotional information in the pictures, either on their own or as cued by us. Because gaze direction moderated some CR effects, subsequent hypothesis testing analyses retained gaze direction contrasts and their interaction terms as fixed effects within subjects.

### Primary analyses

To test our hypothesis that higher cognitive ability would predict more successful CR, we evaluated a two-level structural equation model (referred to as the Cognitive Ability Model in Table 3).

**Table 3:** **Parameter estimates from hypothesis tests examining cognitive ability or age group as predictors of cognitive reappraisal success**.

**Fluid cognitive ability latent variable**	**Cognitive ability model**		**Age group model**		**Expanded model**	
	***B* (*SE*)**		***B* (*SE*)**		***B* (*SE*)**	
Indicators	Factor loadings				Factor loadings	
BD	7.811 (2.305)^*^		–		10.607 (1.274)^**^	
CD	11.039 (3.317)^*^		–		12.688 (2.22)^**^	
DS	1.63 (0.899)^+^		–		2.31 (0.767)^*^	
**Between-subjects predictors**	**Decrease—view criterion**	**Decrease—view criterion**	**Decrease—view criterion**	**Increase—view criterion**	**Decrease—view criterion**	**Increase—view criterion**
Fluid cognitive ability	−0.367 (0.08)^**^	0.431 (0.128)^*^	–	–	−0.423 (0.107)^**^	0.479 (0.152)^*^
Crystallized cognitive ability (VC)	0.007 (0.007)	−0.004 (0.01)	–	–	0.006 (0.008)	−0.002 (0.011)
Age group (older = 1, younger = 0)	–	–	0.207 (0.13)	−0.226 (0.18)	0.102 (0.197)	−0.388 (0.239)
Ever married (yes = 1, 0 = no)	–	–	–	–	−0.558 (0.159)^**^	0.909 (0.217)^**^
Education level (higher values = more educated)	–	–	–	–	−0.012 (0.079)	0.062 (0.096)

Within-subjects effects recapitulate effects reported in Table 2 and thus are not reported here. BD, Block Design; CD, Coding; DS, Digit Span.

** *p < 0.001*,

* *p < 0.05*,

+ p < 0.10.

On the first level, the predictor and criterion variables were the same as noted for the preliminary analysis that tested the main and interactive effects of CR and gaze direction^2^.

On the second level, the slopes estimated from the decrease and increase CR predictors on the first level were treated as criterion variables that were regressed on two between-subjects predictors, fluid cognitive ability and crystallized cognitive ability. Fluid cognitive ability was modeled as a latent predictor variable with three manifest indicators, BD, CD, DS; variance was constrained at 1. Crystallized cognitive ability was modeled as the stand-alone manifest indicator, VC.

As shown in Table 3, factor loadings for the fluid cognitive ability latent predictor variable were positive and significant or nearly so (*p* = 0.001 for BD and CD; *p* = 0.070 for DS). Note that negative estimates of the association between cognitive ability and CR success are expected for the decrease effect since the contrast was calculated as decrease minus view. Positive estimates are expected for the increase effect since the contrast was calculated as increase minus view. Consistent with our hypothesis, this analysis revealed that higher fluid cognitive ability was associated with greater success at decreasing (*B* = -0.37, *p* < 0.001) and increasing (*B* = 0.42, *p* = 0.001) emotional responding. Crystallized cognitive ability as indexed by VC was not associated with the decrease or increase CR effect (both *p* > 0.30).

In follow-up analyses, we repeated the analyses above, but this time specified the fluid cognitive ability latent predictor variable with just one indicator, setting its residual variance and the slopes for the remaining two indicators to 0. In these analyses, higher levels of CD on its own were associated with greater success at decreasing (*B* = −0.19, *p* = 0.023) and increasing (*B* = 0.24, *p* = 0.058, a trend) emotional responding. Similarly, higher levels of BD on its own were associated with greater success at decreasing (*B* = −0.80, *p* = 0.081, a trend) and increasing (*B* = 0.07, *p* = 0.033) emotional responding. Neither of these associations was significant when considering DS on its own (both *p*s > 0.10). In another follow-up analysis, we dropped the latent fluid cognitive ability predictor variable and instead estimated a random effect for each of the four indicators of cognitive ability simultaneously. In this analysis, none of the four indicators was uniquely associated with CR success. Together, these results suggest that it is variance shared primarily between perceptual reasoning (BD) and PS (CD)—putatively fluid cognitive ability—that predicts successful CR.

### Secondary analyses

In pursuit of our secondary goal, we next present analyses that examine whether there are age differences in cognitive ability and CR success.

#### Age differences in cognitive ability

To examine whether the older and YA in this sample exhibited differences in fluid and/or crystallized cognitive ability as in previous studies, we conducted a between-subjects structural equation model. Fluid cognitive ability was estimated as a latent variable with BD, CD, and DS as manifest indicators; crystallized cognitive ability was estimated as the manifest indicator, VC. Both were regressed on age group (1 = older, 0 = younger). This structural equation model fit the data well, χ^2^(4, *N* = 60) = 1.40, *p* = 0.844, RMSEA = 0.00, 90% CI [0.00, 0.10], CFI = 1.00, SRMR = 0.019.

Results confirmed significant positive factor loadings for all three manifest indicators of the fluid cognitive ability latent variable (all *p* < 0.01). In addition, higher fluid cognitive ability was associated with higher crystallized cognitive ability (β = 0.51, *p* = 0.002). Importantly, although older age was not significantly associated with crystallized cognitive ability (β = −0.08, *p* = 0.525), older age was significantly associated with lower fluid cognitive ability (β = −0.89, *p* < 0.001).

#### Age differences in CR success

In light of the preceding analysis indicating a high degree of shared variance between age group and fluid cognitive ability, we first treated older age as the sole between-subjects predictor of emotional responding in a two-level model (referred to as the Age Group Model in Table 3).

On the first level, the predictor and criterion variables were the same as noted for the preliminary analysis that tested the main and interactive effects of CR and gaze direction.

On the second level, the slopes estimated from the decrease and increase CR predictors on the first level were treated as criterion variables that were regressed on one between-subjects predictor, age group (older = 1, younger = 0).

**Figure 2:**
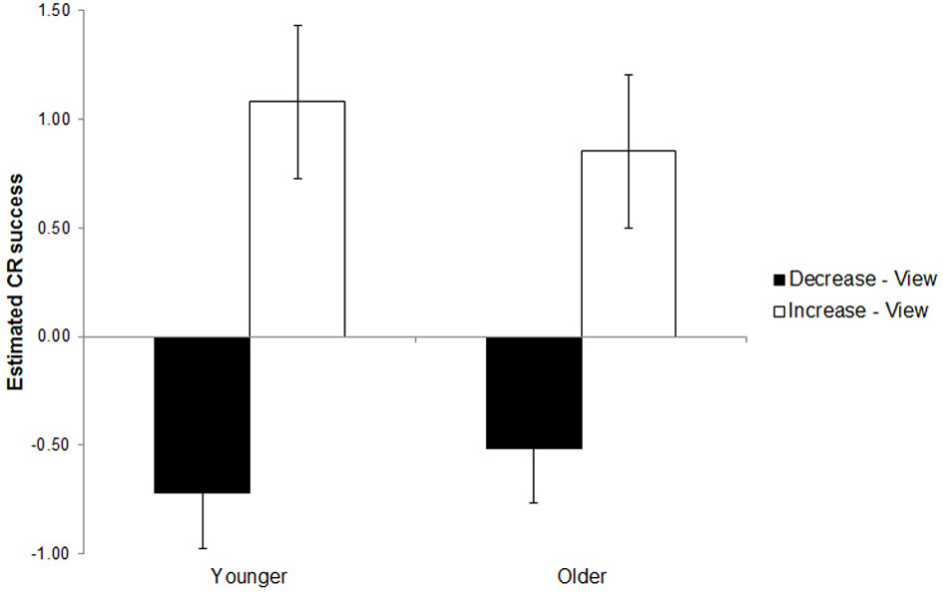
**This figure depicts success using cognitive reappraisal to decrease (black bars) and increase (white bars) emotional responding as a function of age group in the Age Group Model (see Table 3)**. Note that larger negative parameter estimates signal greater success for decrease vs. view whereas larger positive parameter estimates signal greater success for increase vs. view. Success scores were modestly lower in magnitude for older adults (right) than younger adults (left) but neither age difference was statistically significant. Error bars reflect the 95% confidence interval around the estimate of the age difference in cognitive reappraisal (CR) success.

Relative to the moderating effect of fluid cognitive ability, the moderating effect of age group on using CR to decrease (*B* = 0.21, *p* = 0.112) and increase (*B* = −0.23, *p* = 0.209) emotional responding relative to the view condition were modest in magnitude—roughly half the magnitude of the effects reported for fluid cognitive ability. The directions of these effects are consistent with older age predicting reduced CR success (see Figure 2) but they were not statistically significant. Follow-up analyses assessing the age difference within each gaze direction condition revealed effects that also were not statistically significant (results not shown; all *p*s > 0.10).

#### Age differences in CR success: expanded model

It was possible that age differences in CR success were being obscured by individual differences in variables that covaried with age group (fluid cognitive ability, marital status, and education level). Thus, next we examined several simultaneous between-subjects predictors of emotional responding in a two-level structural equation model (referred to as the Expanded Model in Table 3)^3^.

On the first level, the predictor and criterion variables were the same as noted previously.

On the second level, the slopes estimated from the decrease and increase CR predictors on the first level were treated as criterion variables. This time, they were regressed on five between-subjects predictors as follows: fluid cognitive ability, crystallized cognitive ability, age group (older = 1, younger = 0), ever married (1 = yes, 0 = no), and education level.

In this analysis, higher fluid cognitive ability continued to be associated with greater success at decreasing (*B* = −0.42, *p* < 0.001) and increasing (*B* = 0.48, *p* = 0.002) emotional responding. There remained no significant effect of age group on using CR to decrease (*B* = 0.10, *p* = 0.606) or increase (*B* = −0.39, *p* = 0.105) emotional responding relative to the view condition. Notably, relative to when age group was modeled on its own, the magnitude of the effect of age group on using CR to decrease emotional responding was smaller by about one-half; the magnitude of the effect of age group on using CR to increase emotional responding was bigger by about one-half (and just barely approached marginal significance). Interestingly, ever having been married was associated with greater success using CR to decrease (*B* = −0.56, *p* < 0.001) and increase (*B* = 0.91, *p* < 0.001) emotional responding. Neither crystallized cognitive ability nor education level predicted success at decreasing or increasing emotional responding (see Table 3).

#### Estimates of emotional responding during the regulation period on a measure-by-measure basis

The primary and secondary analyses reported above focused on variation in emotional responding during the regulation period modeled as a latent variable with four indicators. Some readers may be interested to examine estimates for each indicator individually as a function of age group, CR instruction, and gaze direction. To produce these estimates, we computed a series of 24 two-level models.

On the first level, there were four criterion variables (self-reported emotional intensity, corrugator activity, HR, EDA during the regulation period). These criterion variables were regressed on three within-subjects, dummy-coded CR instruction predictors. The physiological criterion variables were each additionally regressed on activity during the reactivity period.

On the second level, there were no explicit between-subjects predictors.

In Table 4, we report the B and SE for the intercept, which represents the mean value for the one CR instruction that was not included as a predictor in each model.

**Table 4:** **Estimates of emotional responding for each measure as a function of Cognitive Reappraisal (CR) instruction, gaze direction, and age group**.

	**Younger adults**				**Older adults**			
	**Decrease**	**View**	**Increase**	**No CR**	**Decrease**	**View**	**Increase**	**No CR**
**CORRUGATOR ACTIVITY**								
Arousing	1.267 (0.462)	1.809 (0.517)	2.047 (0.504)	1.956 (0.598)	1.736 (0.47)	2.431 (0.636)	2.984 (0.612)	2.054 (0.453)
Not directed	1.479 (0.539)	2.247 (0.613)	2.37 (0.594)	2.362 (0.667)	1.199 (0.479)	1.8 (0.755)	1.839 (0.542)	1.603 (0.554)
Non-arousing	1.11 (0.34)	1.339 (0.334)	2.018 (0.478)	1.536 (0.37)	1.188 (0.543)	1.404 (0.558)	1.245 (0.457)	1.344 (0.486)
**HEART RATE**								
Arousing	35.424 (3.181)	35.467 (3.048)	35.992 (3.139)	35.636 (3.171)	29.447 (3.494)	29.534 (3.442)	29.62 (3.443)	29.471 (3.449)
Not directed	33.499 (3.807)	33.685 (3.886)	33.728 (3.794)	33.476 (3.847)	32.764 (3.079)	32.866 (3.026)	32.908 (3.001)	32.849 (3.021)
Non-arousing	36.299 (2.659)	36.512 (2.666)	37.335 (2.576)	36.46 (2.541)	29.457 (3.497)	29.482 (3.475)	29.506 (3.469)	29.485 (3.48)
**ELECTRODERMAL ACTIVITY**								
Arousing	−0.015 (0.012)	−0.026 (0.018)	0.002 (0.01)	−0.003 (0.012)	0.004 (0.007)	0.003 (0.006)	−0.003 (0.005)	0 (0.003)
Not directed	−0.007 (0.011)	−0.015 (0.009)	0.002 (0.015)	0.007 (0.012)	0.007 (0.003)	0.007 (0.005)	0.007 (0.003)	0.004 (0.003)
Non-arousing	0.018 (0.01)	−0.001 (0.007)	0.019 (0.011)	0.015 (0.01)	0.001 (0.005)	−0.004 (0.004)	0.003 (0.004)	−0.002 (0.004)
**INTENSITY RATING**								
Arousing	3.658 (0.204)	4.293 (0.238)	5.412 (0.243)	3.967 (0.249)	4.111 (0.28)	4.6 (0.246)	5.771 (0.296)	4.753 (0.282)
Not directed	3.481 (0.203)	4.104 (0.271)	5.491 (0.226)	4.208 (0.236)	4.359 (0.277)	4.655 (0.274)	5.701 (0.273)	4.646 (0.249)
Non-arousing	3.562 (0.206)	3.728 (0.224)	5.136 (0.261)	3.745 (0.235)	4.343 (0.244)	4.371 (0.268)	5.548 (0.3)	4.523 (0.272)

Estimates were determined via a series of two-level multilevel models, one for each age group, gaze direction, and CR instruction as described in text. Reported here is the intercept B(SE) from each model, which represents the mean value for the one CR instruction in each model that was not included as a predictor.

## Discussion

The SOC-ER framework suggests that people might be most successful regulating their emotions when they use strategies for which they have the prerequisite resources in a given situation (Urry and Gross, 2010; Opitz et al., 2012b). In this study, we assessed the role of one class of resources, cognitive ability, in the success of one ER process, CR. Specifically, we examined whether higher levels of cognitive ability would predict greater CR success.

Three important observations emerged from this effort. First, higher levels of fluid cognitive ability predicted greater success at using CR to regulate emotional responses to sad stimuli. Second, fluid cognitive ability predicted greater success at using CR irrespective of whether the regulatory goal was to increase or decrease the emotional response. Finally, despite age differences in cognitive ability in this sample, older age was not associated with variation in success at using CR. We discuss these results in turn below. We then consider broader implications of this work, limitations, and future directions.

### Cognitive ability predicts successful CR

Based on existing neuroimaging studies (see Buhle et al., 2013, for a meta-analysis) and the recent work of Schmeichel et al. (2008), McRae et al. (2012), and Malooly et al. (2013), we tested the prediction that cognitive abilities would predict CR success. Although correlational data preclude causal inference, the present findings are consistent with the idea that fluid cognitive abilities are resources for successful CR. Our results thus support a key tenet of the SOC-ER framework, namely that ER strategies draw on resources—internal abilities and/or environmental affordances that promote the use of a given ER strategy.

Not all previous studies have supported the idea that cognitive abilities should be associated with ER. For example, Farrelly and Austin (2007) failed to observe associations between cognitive ability and “managing emotions” as measured by the Mayer-Salovey-Caruso Emotional Intelligence Test (MSCEIT), the subscale most closely aligned with ER as conceptualized herein. In addition, Gross and John (2003) did not observe correlations between typical use of CR, as measured using the self-report Emotion Regulation Questionnaire (ERQ) and tests of scholastic performance and intelligence. Why, then, did we observe associations between fluid cognitive ability and CR success in this study?

One explanation pertains to the conceptualization and measurement of ER. While the MSCEIT and ERQ tap important aspects of ER, neither assesses success in modulating evoked emotional responses in accord with the emotion-regulatory goal. This is important because generally knowing the best emotion-regulating action to use in a particular context (which is assessed by the MSCEIT) or typically choosing to use CR (which is assessed by the ERQ) may be independent of how well one actually changes their emotional response in the midst of an emotion-triggering situation. Fluid cognitive abilities may be particularly relevant to how well one actually changes one's emotional response in accordance with the regulatory goal in an emotion-triggering situation, particularly in the face of a time limit and complex visual information to appreciate as was the case in this study.

### Cognitive ability predicts successful CR, no matter the goal

Much ER research has focused on the down-regulation of negative emotions. This approach is motivated by the idea that prohedonic regulatory goals (e.g., “decrease negative”) are commonly pursued in daily life (Gross et al., 2006; Riediger et al., 2011), and may have important clinical implications (Goldin et al., 2012; Gross, 2013). However, research focusing on the up-regulation of negative emotions is also warranted for two reasons. First, people also pursue contrahedonic regulatory (e.g., “increase negative”) goals (Riediger et al., 2009, 2011; Tamir et al., 2013). Second, manipulating both pro- and contrahedonic ER in the same study helps to disambiguate the cognitive effort required to reappraise from changes in emotional arousal (see, e.g., Urry et al., 2009).

Examining both pro- and contra-hedonic goals in the present study gave us the opportunity to determine whether success in achieving these two ER goals is differentially predicted by cognitive ability. The existing literature provides mixed suggestions about these potentially differential relationships. On the one hand, prior work suggested that prohedonic ER goals may be more difficult to achieve than contrahedonic ER goals (Ochsner et al., 2004). This implies that fluid cognitive ability should be more useful when decreasing negative emotion than when increasing negative emotion. On the other hand, evidence also suggests that the association between working memory—one cognitive resource—and preferences for contrahedonic emotions is stronger than the association between working memory and preferences for prohedonic emotions (Riediger et al., 2011). This implies that fluid cognitive ability should be *less* useful when decreasing negative emotion than when increasing negative emotion.

Contrary to both possibilities, we found no difference in the extent to which higher levels of fluid cognitive ability predicted successful CR in pursuit of contrahedonic vs. prohedonic goals. Of course, it is possible that features of our design facilitated this outcome. For example, the use of mildly intense sad stimuli and a pseudo-block design may have reduced the typical disparity in difficulty. Future studies will be needed to understand the conditions that moderate how resources like fluid cognitive ability contribute to differential success of ER strategies as a function of contrahedonic or prohedonic aims.

### Age differences in cognitive ability but not CR success

Theoretical models suggest that we might expect age differences in emotion and/or its regulation, at least in some contexts (e.g., Socioemotional Selectivity Theory Carstensen et al., 1999, Strength and Vulnerability Integration Charles, 2010). Indeed, consistent with that expectation, Opitz et al. (2012a) previously demonstrated that OA were less successful than YA at using CR to decrease negative emotions, but more successful at using CR to increase negative emotions. OA in that study also exhibited reduced CR-related activation in brain regions implicated in cognitive control processes like information selection and conflict monitoring compared to YA. The authors concluded that OA may lack the cognitive resources to reduce negative emotion using the particular form of CR that was studied. However, it is important to acknowledge that some of the stimuli used in that study may have been less relevant to OA. Moreover, participants had to frequently switch between ER goals and the regulation period was very brief. That particular design may therefore have put OA at a disadvantage in applying CR.

In the current study, we used only sad, moderately intense pictures which had face-valid relevance to both younger and OA. Additionally, participants had more time to use CR and only had to switch between one active CR condition and the view and no CR control conditions within each block. These changes likely reduced the degree to which OA would be at a disadvantage relative to YA in being able to successfully apply CR. With these changes in place, we observed significant associations between fluid cognitive ability and CR success but not between age group and CR success. Of course, this may in part be because of insufficient power to detect small effects in the present study. However, alternatively or in addition, the age differences in CR success observed by Opitz et al. (2012a) based on fewer participants may have been due to age-related variation in cognitive ability as well as age-related inequality in opportunity for success. Together, these two studies are consistent with the idea that age differences in affective responding are apt to be observed only in “highly resource-demanding situations that overtax OA capacities” (Wrzus et al., 2013, p. 386).

It is important to note that our ability to examine age differences in CR success in this study was hampered for at least two reasons. For one, the age range in the older and younger samples differed (16-year span vs. 4-year span, respectively). The larger age range in the older adult group may introduce greater within-group variation, thus reducing sensitivity to between-group differences. In addition, the two age groups differed not just in terms of age but also in terms of education, marital status, and fluid cognitive ability; OA had more education, were more likely to have been married, and had lower fluid cognitive ability than YA. Thus, even if we had identified age differences in CR success, it would ultimately be unclear whether age itself or one or more of these other variables represents the source of those differences. Importantly, when we modeled all of these as simultaneous predictors in one model, we did not observe any statistically significant effects of age group; instead, ever having been married was associated with greater CR success. Although intriguing, we are reluctant to make inferences about these marital status effects (and the absence of age effects) because none of the YA had ever been married and only three of the OA had never been married; as such, marital status is confounded with older age in this sample. It remains for future research to determine whether ever having been married really does relate to greater CR success, as potentially suggested by our results.

We did not have *a priori* predictions about age differences in the relation between cognitive ability and CR success. Even if we did, as noted above, age was significantly associated with fluid cognitive ability, marital status, and education level in the present sample, which hampers our ability to isolate unique effects. Thus, we suggest that fluid cognitive ability was perhaps a resource for successful CR across the whole sample. We also speculate that the inclusion of older and YA in our sample increased the range of meaningful variation in fluid cognitive ability; this may have enhanced sensitivity to the association between fluid cognitive ability and CR success.

### Limitations and directions for future research

While this study had numerous strengths, including the multi-method approach to measuring emotional responding, inclusion of both younger and older adult participants, and use of multi-level structural equation modeling to test our hypothesis, we acknowledge that this study has some important limitations to be addressed in future research. First, we concentrated on CR, which is just one of five families of ER processes. While individual differences in fluid cognitive ability predict the success of CR, this should not be taken to indicate that fluid cognitive ability would similarly predict the success of other ER strategies. This should also not be taken to indicate that fluid cognitive ability, as indexed by perceptual reasoning, PS, and working memory, are the only resources for CR. By the same token, the present study used only sad picture stimuli. It is unclear whether the current results will generalize to other specific emotions, like fear, disgust, or anger, or to positive emotions like happiness. It will be important in future studies to determine whether the cognitive abilities studied here serve as resources for other families of ER, to identify other resources that support successful CR, and to determine whether these resources play similar roles in the regulation of emotions other than sadness.

Second, cognitive ability is largely internal to an individual. Other resources that might be involved in CR might include resources that are largely *external*. Previous work has proposed, for example, that having access to people who help one generate new meanings for emotion-triggering situations may be a resource for CR (Urry and Gross, 2010). The elegant work of Coan et al. (2006) has demonstrated that stress-sensitive brain regions responded less in women who held someone's hand while undergoing threat of shock compared to women who did not hold someone's hand. While that example is not specific to CR, it suggests that social support in the form of hand-holding may be an external resource that facilitates ER. Assessing external resources for ER is an important direction for future research.

Third, our sample was mostly Caucasian and there were not enough participants to assess potential gender differences in the relations we have reported. In addition, these are cross-sectional, correlational data, which preclude drawing inferences about the putative causal direction on which we have focused here—namely that resources contribute to regulatory success. In order to determine the generalizability of these findings, it will be important to introduce heterogeneity with respect to the types of participants who are recruited for future studies. In addition, to facilitate causal inference, it will be important to (1) measure resources and regulatory success at multiple time points to examine whether a change in resources precedes a change in regulatory success and/or (2) manipulate the resources to see whether a change in resources effects a change in regulatory success.

## Conclusion

In this paper, we provide evidence that higher levels of fluid cognitive ability predict greater success at using CR to increase and decrease sad emotion. This finding supports a basic tenet of the SOC-ER framework (Urry and Gross, 2010), which proposes that higher levels of relevant resources contribute to greater ER success. Importantly, the relation between cognitive ability and successful CR was invariant with respect to whether the goal of CR was contrahedonic or prohedonic. This paper sets the stage for research that seeks to understand variation in successful ER in the context of fluctuating resources. Of key interest from the perspective of SOC-ER is determining the ways in which we compensate for lost resources by (a) selecting different strategies that do not require lost resources and/or (b) optimizing our skill at using strategies for which resources have been compromised. We believe this is an important set of steps to take to further our basic understanding of emotion and its regulation and to further applied goals vis-à-vis augmenting well-being.

## Author contributions

Philipp C. Opitz and Heather L. Urry conceived and designed the study, with significant input from James J. Gross. Philipp C. Opitz collected most of the data. Philipp C. Opitz, Ihno A. Lee, and Heather L. Urry analyzed the data. Philipp C. Opitz, Ihno A. Lee, James J. Gross, and Heather L. Urry wrote the manuscript.

### Conflict of interest statement

The authors declare that the research was conducted in the absence of any commercial or financial relationships that could be construed as a potential conflict of interest.

## Footnotes

^1^Before and after the WAIS-IV subtests reported here, participants completed items assessing appraisals of threat and challenge and current emotional state. Immediately after the CR task, participants provided open-ended reports of strategies used, and items assessing satisfaction with life, state and trait anxiety, ER, perceived stress, health, quality of life, and socioeconomic status. Participants then received information related to a between-subjects stereotype threat manipulation and completed the second set of WAIS-IV subtests (matrix reasoning, letter-number sequencing, symbol search, and the second, difficulty-matched VC subtest). Before and after the second set of WAIS-IV subtests, participants again completed items assessing appraisals of threat and challenge and current emotional state. At the end of the session, participants completed items assessing test anxiety, beliefs about the malleability of intelligence and emotions, age-related stereotypes of cognitive performance, and thoughts about what the experiment was about.

^2^The no CR vs. view contrast is retained in all models. We focus in text on comparisons between the increase and decrease conditions vs. the view condition. We do so because the view condition is a better control than the no CR condition because the view, increase, and decrease conditions all include an auditory instruction on each trial whereas the no CR condition does not.

^3^Because of shared variance between predictors, estimates of the association between each predictor and the criterion variables should be interpreted with caution. In addition, although we attempted to test the interaction between age group and cognitive ability, this model would not converge, even when orthogonalizing cognitive ability with respect to age group.
